# A case of retroperitoneal abscess secondary to duodenal perforation

**DOI:** 10.1093/jscr/rjad368

**Published:** 2023-06-22

**Authors:** Landry Umbu, Hailey Harrison, David Thomas, Megan Contreras, Kwesi Darku

**Affiliations:** Department of Surgery, Trumbull Regional Medical Center, Warren, OH 44483, USA; American University of Antigua, College of Medicine, New York, NY 10005, USA; Department of Surgery, Sharon Regional Medical Center, Sharon, PA 16146, USA; American University of Antigua, College of Medicine, New York, NY 10005, USA; American University of Antigua, College of Medicine, New York, NY 10005, USA

## Abstract

The development of a retroperitoneal abscess in the setting of duodenal perforation is a rare occurrence. There are various causes of duodenal perforation such as trauma, iatrogenic injury and, most commonly, peptic ulcer disease [1]. Urgent surgical intervention is required when a patient presents with a perforated duodenal ulcer and signs of peritonitis. Generally, closure is performed with an omental pedicle or Graham patch [2]. In cases of large perforations, surgical resection, gastric partition with diverting gastrojejunostomy or T-drain placement may be required [2]. In this case, we present a patient with duodenal ulcer perforation complicated by retroperitoneal abscess formation. Treatment involved interventional radiological (IR) drainage of the abscess, followed by laparotomy for persistence of fluid. The surgery comprised of a right-side hemicolectomy, Braun jejunojejunostomy, pyloric exclusion, intraoperative retroperitoneal abscess drainage and Graham patch repair of retroperitoneal duodenal perforation.

## INTRODUCTION

The development of a retroperitoneal abscess in the setting of duodenal perforation is a rare occurrence. There are various causes of duodenal perforation such as trauma, iatrogenic injury, and most commonly, peptic ulcer disease. Urgent surgical intervention is required when a patient presents with a perforated duodenal ulcer and signs of peritonitis. Generally, closure is performed with an omental pedicle or Graham patch. In cases of large perforations, surgical resection, gastric partition with diverting gastrojejunostomy or T-drain placement may be required. In this case, we present a patient with duodenal ulcer perforation complicated by retroperitoneal abscess formation. Treatment involved interventional radiology’s (IR) drainage of the abscess, followed by laparotomy for persistence of fluid. The surgery consisted of a right-side hemicolectomy, Braun jejunojejunostomy, pyloric exclusion, intraoperative retroperitoneal abscess drainage and Graham patch repair of retroperitoneal duodenal perforation.

## CASE PRESENTATION

**Figure 1:**
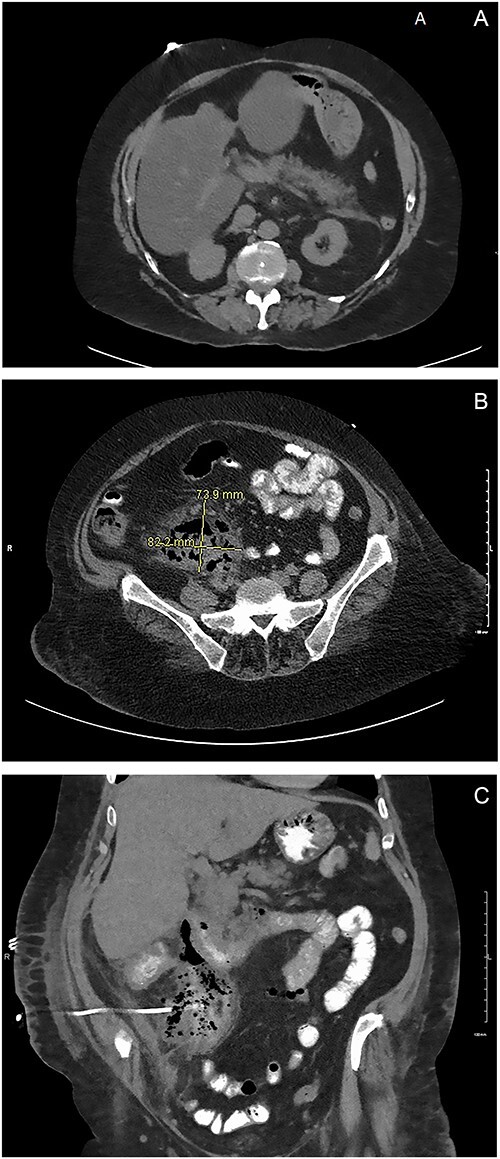
(**A**) ED CT scan demonstrating fat stranding around the pancreas. (**B**) Large fluid collection in the retroperitoneum. (**C**) Oral contrast noted in the IR drain. Large fluid collection in the retroperitoneum consistent with duodenal perforation.

This case presents a 57-year-old female with a past medical history of nicotine dependence, and modified graham patch repair for perforated duodenal ulcer in 2013. She was admitted to the emergency department (ED) complaining of diffuse 10 out of 10 abdominal pain, associated with nausea, vomiting and hematochezia for 2 days. During the examination, it was observed that the patient’s vital signs were stable, and there was tenderness in the epigastric area, with no signs of peritonitis. A computed tomography (CT) scan in the ED showed fat stranding around the pancreas and fluid in the right retroperitoneum consistent with acute pancreatitis (Fig. 1). She was admitted for management of acute pancreatitis. Despite hospital management for 7 days, she continued to have worsening sepsis and blood culture grew anaerobic gram-negative rods.

On hospital day six, general surgery was consulted for evaluation of anemia, with a hemoglobin declining to 6.6. She was given two units of packed RBC and scheduled for upper endoscopy (EGD). The EGD was done on hospital day 10, demonstrating one duodenal ulcer in the first portion of the duodenum. The ulcer was not actively bleeding and showed no signs of inflammation or fistula in the stomach or duodenal. The following day, the patient continued to have worsening leukocytosis. A subsequent CT of the abdomen and pelvis with oral contrast showed a 7 cm × 8 cm multiloculated fluid collection in the retroperitoneum extending from the duodenum to the pelvis. Additionally, a small amount of contrast extravasation was visible around the duodenum consistent with a duodenal perforation in the second or third portion (Fig. 1B). On examination, vital signs were stable, and the abdominal exam was benign. Due to the patient being stable, we elected to proceed with non-operative management. The IR team attempted to drain the fluid collection without success and patient remained septic. A CT abdomen and pelvis with oral contrast was obtained three days later showed a persistent retroperitoneal abscess extending from the level of the duodenum into the upper pelvis. There was evidence of a novel fistulous communication between the abscess and the posterior wall of the descending duodenum suggestive of a perforated duodenal ulcer. Additionally, diffuse wall thickening of the cecum and ascending colon was observed, accompanied by peri-colonic and peri-abscess fat stranding in the surrounding area (Fig. 1C). At this point, surgical exploration was then recommended as a course of action.

A laparotomy incision was performed, revealing significant adhesion of the omentum and small bowel to the abdominal wall. Subsequently, an evaluation of the right colon uncovered a notable abscess that extended throughout the wall of the cecum and into the thickened and inflamed ascending colon. A right hemicolectomy was performed. The retroperitoneal abscess was drained and evacuated. There was a 1-cm perforation on the posterior second part of the duodenum, that was draining significant bilious output. The duodenal perforation was then repaired using a Graham patch with an omental pedicle flap. Two 19 French Blake drains were placed around the area of duodenal perforation. To protect our duodenal repair, we decided to perform a pyloric exclusion procedure. A Braun jejunojejunostomy was performed to reduce bile reflux. The patient tolerated the procedure well, had an uneventful hospital stay thereafter and tolerated diet advancement. Ultimately, the patient was discharged to a skilled nursing facility for further care.

## DISCUSSION

Retroperitoneal abscess can be primary, resulting from direct hematogenous spread or secondary, from some other infected nearby organ. The causes of secondary retroperitoneal abscess are numerous and differ depending on the origin. Common organs that seed secondary retroperitoneal abscesses are the kidneys, GI organs, nearby bony structures and malignant neoplasms [6]. They can also result from procedures or trauma. In the case of our patient, the preexisting history of peptic ulcer disease, and current ulcer formation, was the initial point of infection that resulted in the formation of the retroperitoneal abscess. Duodenal perforation is most often a result of peptic ulcer disease [6]. Retroperitoneal abscess secondary to duodenal perforation is rare [7].

Retroperitoneal abscess formation secondary to duodenal perforation can be a clinically challenging case to diagnose and manage [1]. Initial imaging should be aimed at CT of the abdomen [5]. The treatment of a retroperitoneal abscess secondary to a duodenal ulcer perforation includes IR drainage, surgical closure of the defect and appropriate antibiotics [7]. Although managing the retroperitoneal abscess is important, treating the underlying cause is also necessary. Different methods can be used depending on the location and size of the perforation. For perforations adjacent to the gastric pylorus, truncal vagotomy with pyloroplasty can be considered. However, most small duodenal perforations in other parts of the duodenum are repaired with a Graham patch. A study that compared morbidity and mortality of patients with duodenal perforation treated by surgical closure versus with stent placement showed no significant difference [3]. Stent placement could be beneficial in the presence of suture-line leakage [3].

To prevent the development of complications, early recognition and treatment are key [5]. A cohort study of 2668 surgically treated patients in Denmark showed that for every hour of surgical delay following admission, there was a 2.4% decrease in a 30 day survival [4]. The patient presented in our case had surgery performed 17 days after admission. This delay in treatment could result in higher probability of complications; hence, the extended hospital stays of over a month. Complications include sepsis, diffuse peritonitis and even death [7].

## CONCLUSION

In this report, we presented a rare case of retroperitoneal abscess in the setting of duodenal perforation. This condition can result in sepsis, diffuse peritonitis and even death if prompt identification and action does not occur. Identifying the source of retroperitoneal abscess can be challenging and some patients can present without abdominal symptoms. Identification with a CT of the abdomen is crucial to diagnosis. Management of a retroperitoneal abscess involves CT guided drainage, antibiotics and possible surgical management of underlying causes. The ideal treatment of retroperitoneal abscess due to duodenal perforation is surgical intervention including laparoscopy, robotic approach or laparotomy. Endoscopic intervention and IR drainage are other possible treatments.

## Data Availability

Data sharing is not applicable to this article as no new data were created or analyzed in this study.

## References

[1] Ansari D, Torén W, Lindberg S, Pyrhönen HS, Andersson R. Diagnosis and management of duodenal perforations: a narrative review. Scand J Gastroenterol 2019;54:939–44. 31353983 10.1080/00365521.2019.1647456

[2] Søreide K, Thorsen K, Harrison EM, Bingener J, Møller MH, Ohene-Yeboah M, et al. Perforated peptic ulcer. Lancet 2015;386:1288–98. 26460663 10.1016/S0140-6736(15)00276-7 PMC4618390

[3] Arroyo Vázquez JA, Khodakaram K, Bergström M, Park PO. Stent treatment or surgical closure for perforated duodenal ulcers: a prospective randomized study. Surg Endosc 2021;35:7183–90. 33258032 10.1007/s00464-020-08158-3 PMC8599331

[4] Buck DL, Vester-Andersen M, Møller MH. Danish clinical register of emergency surgery. Surgical delay is a critical determinant of survival in perforated peptic ulcer. Br J Surg 2013;100:1045–9. 23754645 10.1002/bjs.9175

[5] Altemeier WA, Alexander JW. Retroperitoneal abscess. Arch Surg 1961;83:512–24. 13860719 10.1001/archsurg.1961.01300160024004

[6] Townsend CM, Beauchamp RD, Evers BM, Mattox KL, Christopher F. Abdominal wall, umbilicus, peritoneum, mesenteries, omentum and retroperitoneum. In: Sabiston Textbook of Surgery: The Biological Basis of Modern Surgical Practice, 21st edn. Essay, Elsevier, 2022, 1080–104.

[7] Mao X, Yu N, Jia X, Fan W. Imaging findings and clinical features of atypical retroperitoneal abscess caused by duodenal perforation: a case report and review of the literature. J Med Case Reports 2020;14:105. 10.1186/s13256-020-02393-x PMC7367392 32678002

